# P-1417. High Rates of Patient Directed Discharge Associated with Incomplete Treatment and Rehospitalization in People Hospitalized with Serious Injection Related Infections: The CHOICE+ Cohort

**DOI:** 10.1093/ofid/ofae631.1592

**Published:** 2025-01-29

**Authors:** Elana S Rosenthal, Sarah Kattakuzhy, Habib Omari, Jasmine Stevens, Meghan Derenoncourt, Ishan Kumar Vaish, Hannah E Flores, Ayako Wendy Fujita, Sumitha Raman, Alaina Steck, Irene Kuo, Jillian S Catalanotti, Joseph E Carpenter, Becky Reece, Edward C Traver

**Affiliations:** Institute for Human Virology (IHV), University of Maryland School of Medicine, Washington, District of Columbia; Institute for Human Virology (IHV), University of Maryland School of Medicine, Washington, District of Columbia; University of Maryland Baltimore, Baltimore, Maryland; University of Maryland School of Medicine, Baltimore, Maryland; University of Maryland, Baltimore, Baltimore, Maryland; University of Maryland Medical School, Baltimore, Maryland; University of Maryland, Baltimore, Maryland; Emory University School of Medicine, Atlanta, Georgia; George Washington University, Washington, District of Columbia; Emory University, Atlanta, Georgia; George Washington University Milken Institute School of Public Health, Washington, District of Columbia; The George Washington University of Medicine and Health Sciences, Washington, District of Columbia; Emory University School of Medicine, Atlanta, Georgia; West Virginia University, Morgantown, WV; University of Maryland School of Medicine, Baltimore, Maryland

## Abstract

**Background:**

People who inject drugs have high rates of patient directed discharge (PDD). In a cohort of patients hospitalized for serious injection related infections (SIRI), we aimed to assess the factors leading to PDD and the influence of PDD on rehospitalization.

Characteristics of study participants

**Figure:**
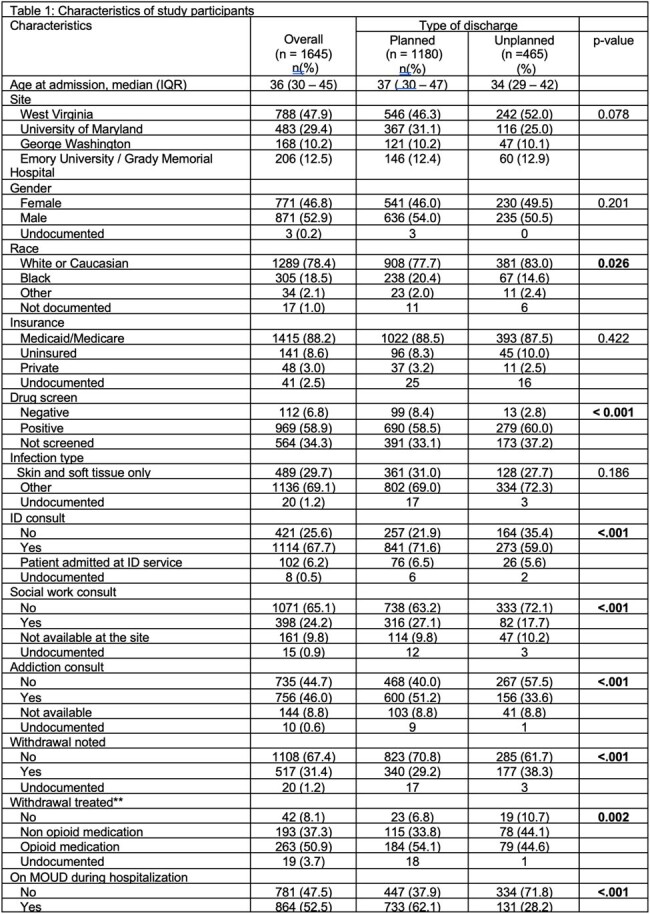

**Methods:**

CHOICE+ is a multisite retrospective cohort study of adults hospitalized at four healthcare systems with SIRI due to injection opioid use between 1/1/2018 and 3/31/2022. Data were collected by abstraction of the electronic medical record and were analyzed by chi-square and multivariable logistic and linear regression.

Factors associated with patient directed discharge

**Figure:**
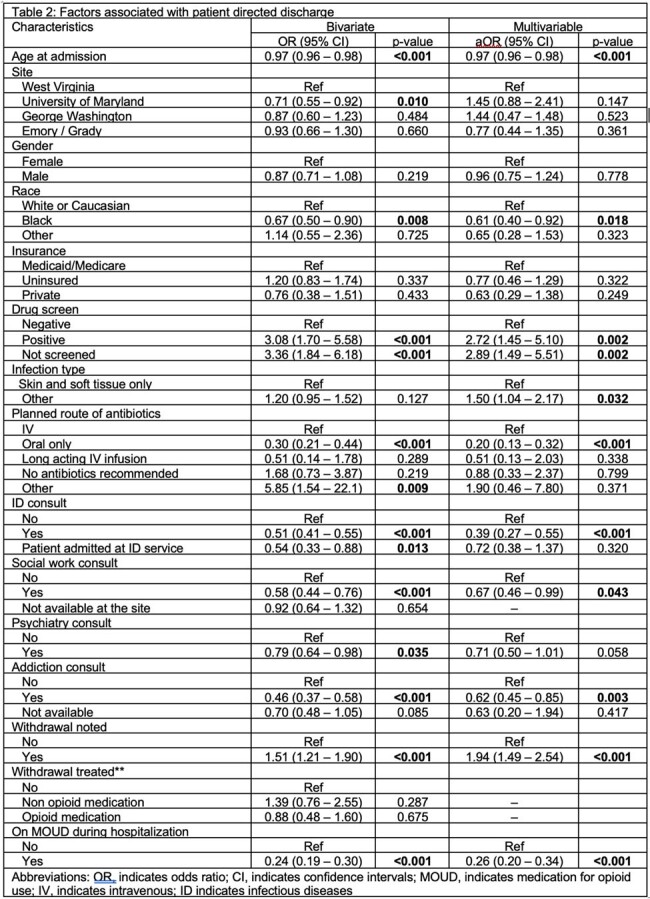

**Results:**

Of 1645 patients, 1180 (72%) had a planned discharge and 465 (28%) had a PDD. PDD was associated with younger age (p< 0.001), white race (p=0.018), and documented opioid withdrawal (p< 0.001). PDD was less likely in patients on medication for opioid use disorder (MOUD) during hospitalization (p< 0.001) or who received consultations by infectious diseases (p< 0.001), social work (p=0.04), or addiction medicine (p=0.003).

Compared to those with planned discharge, patients with PDD were less likely to have completed antibiotics at the time of discharge (7% vs 60%; p< 0.001), and 64% left without a plan for antibiotic completion. Patients with PDD were more likely to be readmitted within a year (57% vs 52%; p=0.015), and more likely to have a first readmission due to an infection (81% vs 56%; p< 0.001). Time to readmission was significantly shorter among patients with PDD (15 vs 65d; -41.5 days, p< 0.001).

Post-Hospitalization Antibiotic Plan by Discharge Status

**Figure:**
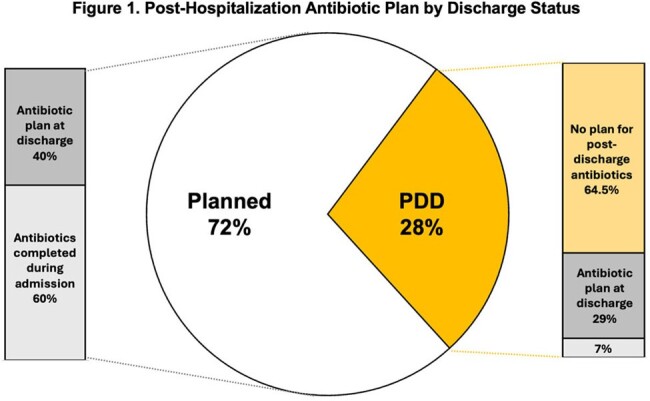

**Conclusion:**

Among people hospitalized with SIRI, we found high rates of PDD associated with insufficient treatment of OUD, evidenced by withdrawal, lack of MOUD, and not receiving an addiction consultation. PDD was consequently associated with higher rates of readmission due to infection and shorter time to readmission, possibly due to discharge without a plan to complete antibiotics. To improve outcomes and reduce readmissions among people with SIRI, efforts to address OUD during hospitalization are crucial, and could reduce PDD. Regardless, given high rates of PDD in this population, strategies to facilitate continuity of antibiotics - including contingency plans for oral or long-acting regimens - may be critical to ensuring resolution of SIRI and improving long-term outcomes.

Risk of Readmission within 1-Year Based on Discharge Status

**Figure:**
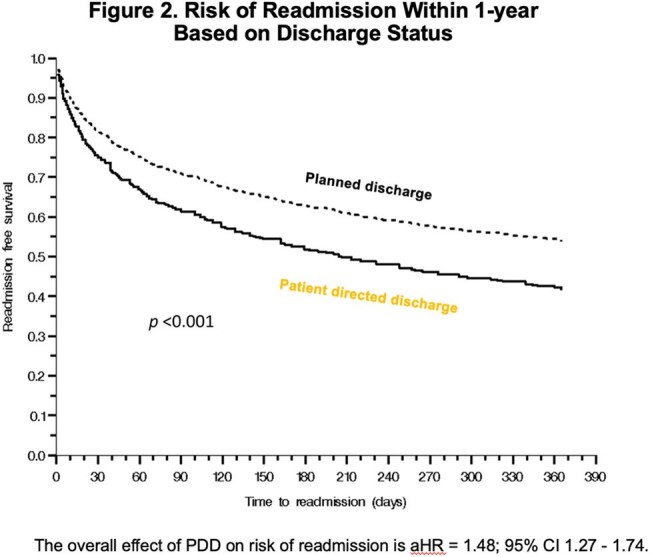

**Disclosures:**

**Elana S. Rosenthal, MD**, Gilead Sciences: Grant/Research Support|Merck: Grant/Research Support

